# Novel Water-in-Oil Emulsions for Co-Loading Sialic Acid and Chitosan: Formulation, Characterization, and Stability Evaluation

**DOI:** 10.3390/foods11060873

**Published:** 2022-03-18

**Authors:** Min Pang, Donglei Zheng, Pengpeng Jia, Lili Cao

**Affiliations:** 1 School of Food Science and Bioengineering, Hefei University of Technology, Hefei 230009, China; pangmin@hfut.edu.cn (M.P.); zheng_donglei@mail.hfut.edu.cn (D.Z.); 2020171465@mail.hfut.edu.cn (P.J.); 2 Key Laboratory for Agricultural Products Processing of Anhui Province, Hefei 230009, China

**Keywords:** water-in-oil (W/O) emulsion, sialic acid, stabilization

## Abstract

This study was designed to co-load sialic acid (SA) and chitosan in a water-in-oil (W/O) emulsion and investigated its characterization and stability. Emulsions were prepared using two different oils (olive oil and maize oil) and polyglycerol polyricinoleate (PGPR) alone or in combination with lecithin (LE) as emulsifiers. The results revealed that the aqueous phase of 5% (*w*/*v*) SA and 2% (*w*/*v*) chitosan could form a stable complex and make the aqueous phase into a transparent colloidal state. Increasing the concentration of PGPR and LE presented different effects on emulsion formation between olive oil-base and maize oil-base. Two stable W/O emulsions that were olive oil-based with 1.5% (*w*/*v*) PGPR+ 0.5% (*w*/*v*) LE and maize oil-based with 2% (*w*/*v*) PGPR+ 0% (*w*/*v*) LE were obtained. Initial droplet size distribution curves of the two stable emulsions displayed unimodal distribution, and the rheological curves displayed the characteristics of shear thinning and low static shear viscosity. Moreover, the storage stability showed that there was no significant change in droplet size distribution and Sauter mean diameter of the emulsions at room temperature (25 °C) for 30 days. These results indicated that the W/O emulsions could effectively co-load and protect sialic acid and chitosan and thus could be a novel method for increasing the stability of these water-soluble bioactive compounds.

## 1. Introduction

The excellent properties of emulsion determine its bright application prospect in the fields of food, medicine, and cosmetics. The dispersion system of emulsion is formed by the dispersion of a liquid as droplets in another liquid that is immiscible with it, and the stability of the emulsion system can be enhanced by using surfactants with proper hydrophilic and lipophilic balance (HLB) [1]. The W/O emulsion is a thermodynamically unstable system because of the high interfacial area of the dispersed phase. The high fluidity of water droplets will promote the instability of W/O emulsion through precipitation flocculation, polymerization, and Ostwald ripening [2]. The stability of water-in-oil emulsion depends on the composition of the emulsion and various process parameters, including the type and concentration of hydrophobic emulsifier [3], nature of the oil phase [4], homogenization parameters [5], the ratio of oil phase to water phase [6], and homogeneous technology [7].

Emulsifiers that can form W/O emulsions need a low HLB. Lecithin is the only natural emulsifier at present, mainly from soybeans and eggs. The principal components of Lecithin include phosphoric acid, choline, fatty acid, glycerol, glycolipid, triglyceride, and phospholipid [8]. Although lecithin is soluble in mineral oil, it cannot be dissolved in unheated vegetable oil. It is also insoluble in water but can be highly dispersed in water [2]. PGPR, which is an oligomeric nonionic surfactant formed by esterification of castor oil fatty acids and polyglycerol, is an extremely strong lipophilic emulsifier [9]. It is used to reduce the viscosity of chocolate products [10]. A suitable emulsifier is very important for the establishment of stable oil-in-water emulsions.

Sialic acid, which was isolated from mucin of the submandibular gland early in 1957 [11], is an acidic aminosaccharide (5-acetamido- 3,5-dideoxy-D-glycerol-D–galacta-nonyl ketone sugars) with nine pyranose structures [12]. Sialic acid has been proven to take part in a variety of physiological functions on the cell surface and plays a very important role in regulating human physiological and biochemical functions. Under physiological conditions, sialic acid has a negative charge, is easy to combine with positively charged minerals and some vitamins (B_12_), and improves their absorption efficiency [13,14]. Sialic acid usually binds to the non-reducing ends of the sugar chains of glycolipids and glycoproteins and plays the role of receptor ligands in fertilization, immunity, differentiation, information transmission, and other activities [15]. Additionally, sialic acid is an important component of gangliosides in the animal brain, which is related to memory ability and intellectual development, and is related to neonatal liver function [16].

The molecule of sialic acid is described as a strong organic acid, and its aqueous solution is acidic since the carboxyl group is in the first position and the amino group is in the fifth position, which produces a negative charge under physiological conditions [17]. Rohrig found that negatively charged sialic acid molecules with high hydrophilicity can pass through the cell membrane [18], and a study in vitro confirmed that isotope-labeled sialic acid could be efficiently absorbed by eukaryotic cells [19]. The biological function of sialic acid is related to its acidic groups, so it is essential to protect them since the acidic groups are easily oxidized when sialic acid is exposed in vitro.

Chitosan is a kind of linear amino-polysaccharide obtained by deacetylation of natural polysaccharide chitin. Chitosan is easily soluble in aqueous acidic media below pH = 6.0 and is used as a water-soluble cationic polyelectrolyte [20]. The aqueous solution of macromolecular chitosan is a typical non-Newtonian pseudoplastic fluid with viscoelasticity and shear thinning properties. It has been reported that the molecular weight, degree of deacetylation, chitosan concentration, and additional salt ion content have great influence on the rheological properties of chitosan aqueous solution [21,22]. Chitosan could be used as a basic skeleton to prepare microcapsules, microspheres, or nanodroplet drug delivery systems because chitosan has good physical and chemical properties, such as biodegradability, biocompatibility, availability, low cost, adhesion, antimicrobial properties, and appropriate modification ability [23,24]. Zhang et al. used chitosan solution as an aqueous phase to prepare W/O emulsion with good stability [25]. The objectives of this study were to establish a novel, stable oil-in-water (W/O) emulsion system for loading sialic acid. Chitosan was added to stabilize the acidic groups of sialic acid. The system developed could effectively separate sialic acid from the environment to avoid the adverse effects of hydrogen ions. The mechanism of the stability of the loading system was expounded, and the properties were characterized and evaluated. This work attempts to provide an in-depth understanding of the stability and technical support for the practical application of sialic acid.

## 2. Materials and Methods

### 2.1. Materials and Chemicals

Sialic acid was provided by Wuhan Zhongke Guanggu Green Biotechnology Co., Ltd. Polyglycerol polyricinoleate (PGPR, the content of diglycerol, triglycerol, and tetramyglycerol ≥ 75 w/%, the content of heptaglycerol ≤ 10 w/%) and chitosan (deacetylation degree is 95%, viscosity is 100–200 mPa·s) were purchased from Macklin biochemical Technology Co., Ltd. (Shanghai, China). Lecithin (LE) was purchased from TCI (Shanghai, China) Development Co., Ltd. Fatty acid standard products was purchased from Sigma Aldrich (Shanghai, China) Trading Co., Ltd. Olive oil and maize oil were bought from a local supermarket. Methanol (HPLC) and n-hexane (HPLC) were purchased from Shanghai Yuesheng Trading Co., Ltd. (Shanghai, China). Boron trifluoride-methanol solution 14% in methanol for esterification of fatty acids for GC purposes was purchased from ANPEL Laboratory Technologies (Shanghai, China) Inc. NaCl (AR) and NaOH (AR) were purchased from National Pharmaceutical Group Chemical Reagent Co., Ltd. (Shanghai, China). Ultrapure water was produced by a Milli-Q system (Millipore, Gradient, UK).

### 2.2. Preparation of Aqueous Phase and W/O Emulsions

Hydrated solution of the aqueous phase of W/O emulsions was prepared by dissolving 5 w/% sialic acid and 2 w/% chitosan (the sialic acid was first dissolved completely, then chitosan was dissolved, because the chitosan could only be dissolved in acidic solutions) in Ultra-pure water while stirring at room temperature (25 °C) until sialic acid and chitosan were completely dissolved, and the solution was in a clear state. Then, the solution was dried by a vacuum freeze dryer until all the water was gone. Vacuum freeze-dried sialic acid and chitosan complex, which presented a platelike state, were ground into powders and kept in the refrigerator at 4 °C for further research.

The W/O emulsion was prepared using the modified procedures described by [26]. The composition of the oil phase includes olive oil, maize oil, 0–2 w/% PGPR, and 0–2 w/% LE. PGPR and LE, which were used as the emulsifier, were previously dissolved into oil. The aqueous phase and oil phase were mixed at 3:7 (15 g aqueous phase, 35 g oil phases) (w_aqueous phase_/w_oil phase_) ratios and stirred at 1500 rpm for 15 min until the mixture reached the initial emulsification state to obtain the crude emulsion. Then, the crude emulsions were homogenized at 22,000 rpm for 3 min. The prepared emulsions were stored at 25 °C for further research.

Emulsion samples Em 1, Em 2, Em 3, Em 4, and Em 5 were prepared as olive oil-based emulsions with 2.0 w/% PGPR, 1.5 w/% PGPR and 0.5 w/% LE, 1.0 w/% PGPR and 1.0 w/% LE, 0.5 w/% PGPR and 1.5 w/% LE, and 2.0 w/% LE, respectively. Emulsion samples Em 6, Em 7, Em 8, Em 9, and Em 10 were prepared as maize oil-based emulsions with 2.0 w/% PGPR, 1.5 w/% PGPR and 0.5 w/% LE, 1.0 w/% PGPR and 1.0 w/% LE, 0.5 w/% PGPR and 1.5 w/% LE, and 2.0 w/% LE, respectively.

### 2.3. High-Resolution Field Emission Scanning Electron Microscope (HRFESEM)

The surface condition of the dry samples was observed by a high-resolution field emission scanning electron microscope (Hitachi Regulus 8230, Tokyo, Japan). The observation voltage was set to 3 kV, and the image was magnified 1000 times.

### 2.4. Fourier Transformed Infrared Spectroscopy (FT-IR)

The structure analysis of the sialic acid and chitosan complex was examined by Fourier transform infrared spectroscopy (FT-IR). The IR spectra of the samples were recorded by an FT-IR spectrophotometer (Thermo Nicolet, Madison, WI, USA). The spectrum was scanned in transmission mode from 400 to 4000 cm^−1^ wavenumber range. The dry samples were blended with KBr powder at a ratio of 100:1 and pressed into a disk before spectrum acquisition.

### 2.5. X-ray Diffraction (XRD)

XRD patterns of sialic acid, chitosan, and complex were measured with an X-ray diffractometer (PANalytical X-Pert PRO MPD, Almelo, The Netherlands). The diffraction angle of 2θ was from 5° to 70°.

### 2.6. Thermogravimetric Analysis (TG)

TG of sialic acid, chitosan, and complex were determined using a thermogravimetric analyzer (TGA8000, PE, Waltham, MA, USA) to determine the thermal stability. The samples were determined under temperatures from 30 to 700 °C at a heating rate of 10 °C/min and switch the gas to nitrogen at 20.0 mL/min.

### 2.7. Microstructure Measurement

The samples, which were taken a little on the glass slide and gently covered with the cover glass, after being diluted to an appropriate multiple by the corresponding oil base, were observed using a microscope (Mshot, ML31, Guangzhou, China) with a 100-fold oil mirror. The images were collected using a microscopic camera (Mshot, MS60. Guangzhou, China) with an interface (Mshot, TV0-5XC, Guangzhou, China).

### 2.8. Droplet Size Distribution

Droplet size distribution of W/O emulsions was measured using Malvern Mastersizer 2000 (Malvern Instruments Ltd., Malvern, UK) based on the methods described by [2] and [27] with slight modifications. The emulsions were dispersed in n-hexane (refractive index 1.375). The measurements were done at laser obscuration between 5% and 10%, and the rotate speed of the circulating pump was set to 2000 rpm. The refractive index and absorption of aqueous phase were 1.344 (measured by Abbe refractometer) and 0.1, respectively. Droplet size data are presented as a volume fraction.

The Sauter mean diameter (*d*_3,2_) was calculated using Equation (1) and used to represent the droplet size.

(1) $$d_{3,2}=\frac{\sum \left(n_{i}\cdot d_{i}^{3}\right)}{\sum \left(n_{i}\cdot d_{i}^{2}\right)}$$

where *n_i_* is the number of droplets with the size of *d_i_*.

The uniformity, a measure of the absolute deviation from the median, was calculated using Equation (2) and used to represent symmetry of droplet size distribution.

(2) $$\mathrm{Uniformity}=\frac{\sum X_{i}\left|d\left(x,\;\;0.5\right)\;-\;d_{i}\right|}{d\left(x,0.5\right)\sum X_{i}}$$

where *d* (*x*, 0.5) is the size of the droplet below which 50% of the samples and *X_i_* is the volume of the number of droplets with diameter *d*_i_ existing between the two consecutive diameters [28].

### 2.9. Rheological Characterization

The rheological properties of W/O emulsions were measured according to the methods described by [29,30] with some modifications using a DHR-3 rheometer (TA Instruments, Leatherhead, UK) equipped with a temperature-controlling system. A cone-and–plate measurement cell (diameter 40 mm, angle 2°) was used for shear-viscosity measurements. The sample was placed on the lower plate, and then the upper cone was lowered and a small amount of sample was injected along the slit using an adjustable-volume pipette to ensure that the sample was contained within the space between plate and cone. The flow behavior of W/O emulsions was measured at 25 °C, and shear rate was increased from 0.01 to 50 s^−1^.

### 2.10. Storage Stability

A 25 mL W/O emulsion was poured into a cylindrical glass sample bottle and stored at room temperature (25 ± 1 °C). The stability of the emulsion was characterized by the sedimentation index (SI) and the variation of droplet size distribution (the test method was given above). The SI represents the amount of phase separation owing to Ostwald ripening, flocculation, or coalescence, and the phase separation of the samples was visually evaluated. SI was defined by Equation (3).

(3) $$\mathrm{SI}\left(\%\right)=\frac{H}{H_{0}}\times 100\%$$

where *H* represents the height of unstable zones, and *H_0_* represents the initial height of emulsion. The value of SI is smaller, and the stability of emulsion is better.

### 2.11. Statistical Analysis

All tests were performed independently in triplicate, and the results were recorded as the mean ± standard deviation (SD). Statistics were analyzed by analysis of variance (ANOVA) with IBM SPSS Statistics 26. Significance was defined at *p* < 0.05 by Tukey’s honestly significant difference (HSD).

## 3. Results and Discussion

### 3.1. HRFESEM of the SA-Chitosan Complex

**Figure 1:**
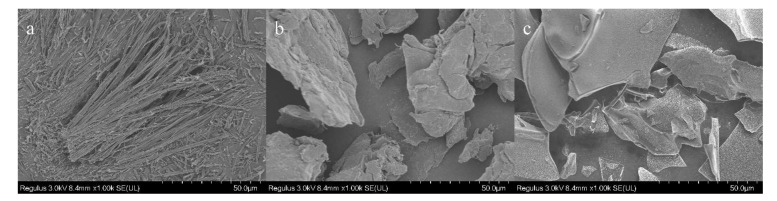
Microscopic morphology of sialic acid (**a**), chitosan (**b**), and 5 w/% sialic acid and 2 w/% chitosan complex (**c**).

The surface texture of the samples is displayed in Figure 1. SA was a rod-like crystal (Figure 1a), and chitosan existed as a block with a rough surface (Figure 1b), while the complex was displayed in the form of a block with a smooth surface (Figure 1c). The surface of the complex in Figure 1c is different from that of sialic acid and chitosan, indicating that sialic acid was bound to chitosan and coated by chitosan, which possibly attributed to the change of the surface caused by the combination of them.

### 3.2. FT-IR Analysis of the SA-Chitosan Complex

**Figure 2:**
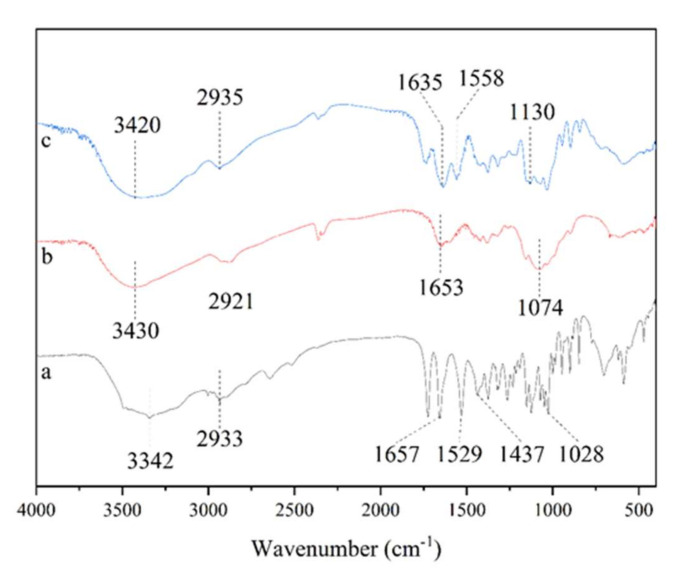
FT-IR spectra of sialic acid (**a**), chitosan (**b**), and 5 w/% sialic acid and 2 w/% chitosan complex (**c**).

The FT-IR spectra of SA, chitosan, and the complex in Figure 2 revealed that the absorption peaks corresponded to the stretching of O-H between 3000 and 3700 cm^−1^ and stretching of C-H around 2935 cm^−1^ [31]. It was acknowledged that O-H is a strongly polar group and extremely easy to associate with other polar groups as hydrogen bonds. It was proved that medium intensity bands in the 3450–3550 cm^−1^ range manifest intermolecular, and 3540–3570 cm^−1^ range bands indicate the presence of some intramolecular hydrogen bonds in Figure 2 [32]. We suspected that the change in the hydroxyl absorption peak could be caused by the formation of hydrogen bonds (intermolecular hydrogen bonds: SA and SA, CH and CH; intramolecular hydrogen bonds: SA and H_2_O, SA and CH, CH and H_2_O). Intermolecular hydrogen bonding is an important factor in maintaining the stability of a complex.

FT-IR spectra of sialic acid, chitosan, and complex within the range of 400–2000 cm^−1^ differed from each other. The spectra of sialic acid (Figure 2a) revealed the presence of characteristic functional groups at 1657 cm^−1^, 1529 cm^−1^, 1437 cm^−1^, and 1028 cm^−1^ corresponding, respectively, to the stretching of C=O, and the bending of N-H, C-H, and C-N bonds [33]. The spectra of chitosan (Figure 2b) contained characteristic absorbance bands at 1653 cm^−1^ and 1074 cm^−1^ corresponding to stretching vibrations of the carbonyl moieties of N-acetylglucosamine links and the vibration of skeleton and C–O–C bond of pyranose cycles, respectively [34]. The spectra of complex (Figure 2c) exhibited a band at 1130 cm^−1^, which is related to the stretching of C=O of sialic acid. Beyond that, the peak was shown at 1558 cm^−1^, which contradicts the characteristic absorption peak of the amide bond [3,35]. This may be due to the shift of the absorption peak caused by the carboxyl group of sialic acid with negatively charged is closely bound to the carboxyl group of chitosan with positively charged under the action of interaction between positive and negative charges. In addition, the peak, which was attributed to stretching of C=O, was shown at 1635 cm^−1^.

The stability of the complex benefits from the interaction of various functional groups of sialic acid and chitosan. Since a new amide group in the complex was not found in FT-IR analysis, it could be speculated that the forces for maintaining the stability of the complex may be the hydrogen bond, the non-amide bond interaction effect between the carboxyl group of sialic acid and the amino group of chitosan, and the three-dimensional network structure formed by chitosan.

### 3.3. Polymorphism of the SA-Chitosan Complex

**Figure 3:**
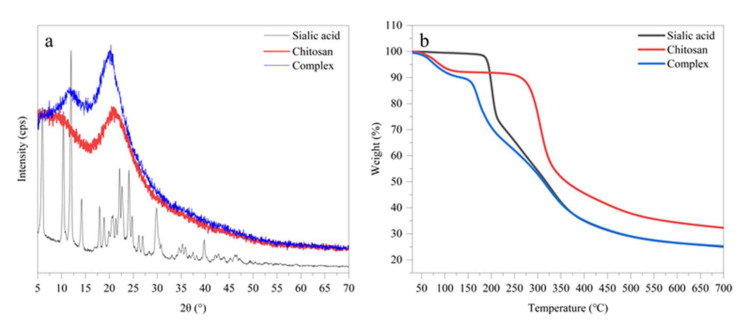
X-ray Diffraction (**a**) and Thermogravimetric Analysis (**b**) of sialic acid, chitosan, and the SA-chitosan complex of 5 w/% sialic acid and 2 w/% chitosan.

Crystal polymorphs of SA, chitosan, and SA-chitosan complex could be evaluated by X-ray diffraction (XRD) patterns based on the short spacing of crystals. Figure 3a shows that SA has sharp diffraction peaks at 2θ = 6.00°, 10.40°, 11.98°, 14.22°, 17.94°, 18.87°, 20.74°, 22.15°, 22.64°, 24.10°, 24.77°, 26.20°, 26.96°, 29.87°, and 39.83°, while chitosan exhibited only one diffraction peak at 20.46°. The SA-chitosan complex exhibited diffraction peaks at 2θ = 11.62° and 20.30°, with the former inheriting from SA and the latter from chitosan. The complex only inherits the diffraction peak of SA at 2θ = 11.98°, which may be caused by the destruction of the original well-organized molecular chain of chitosan and rearrangement due to interaction between positive and negative charges respectively from -NH_2_ of chitosan and -COOH of SA. This finding is consistent with the previous study that the complex formed was displayed in a newly well-organized crystal structure [36].

### 3.4. Thermogravimetric Analysis of the SA-Chitosan Complex

Thermogravimetric analysis for evaluating the thermal stability of SA, chitosan, and SA-chitosan complex and the results are displayed in Figure 3b. The weightlessness of SA is mainly concentrated between 169.29 °C and 700.00 °C. SA lost weight rapidly between 169.29 °C and 212.04 °C, in which the mass decreased from 98.93% to 74.82%, and the rate of weightlessness slowed down between 212.04 °C and 700.00 °C, in which the weight decreased to 24.99%. The weightlessness of SA is due to the degradation of the glucopyranose ring of sialic acid and the functional groups on the pyranose ring [37]. The weightlessness curve of chitosan can be divided into two stages. The first weightlessness stage of chitosan at 30.00–132.96 °C was related to the evaporation of bound water, and the weight decreased to 92.5%. The next weightlessness stage of CH at 226.21–700 °C is mainly caused by the rupture of the major chain of polysaccharides and the degradation of the pyran ring, and the mass decreased from 91.59% to 32.29%. The weightlessness curve of chitosan is consistent with the results observed by Hu et al. [38]. The weightlessness curve of the complex can be divided into two stages. The first weightlessness stage of the complex was at 30.00–155.00 °C, in which the mass decreased to 88.45%, mainly because of the evaporation of bound water in chitosan. The second weightlessness stage of complex at 155.00–700.00 °C, in which the mass decreased to 25.24%, could be attributed to the rupture of the major chain of polysaccharides, the degradation of the pyranose ring, and the destruction of the microstructure combined by interaction between positive and negative charges and intermolecular hydrogen bond. The thermal stability of the composite is worse because the bonds between -NH_2_ of SA and -COOH of CH formed by interaction between positive and negative charges are more easily broken and degraded under high temperature [36].

### 3.5. Optical Microscopy Imaging of W/O Emulsions

**Figure 4:**
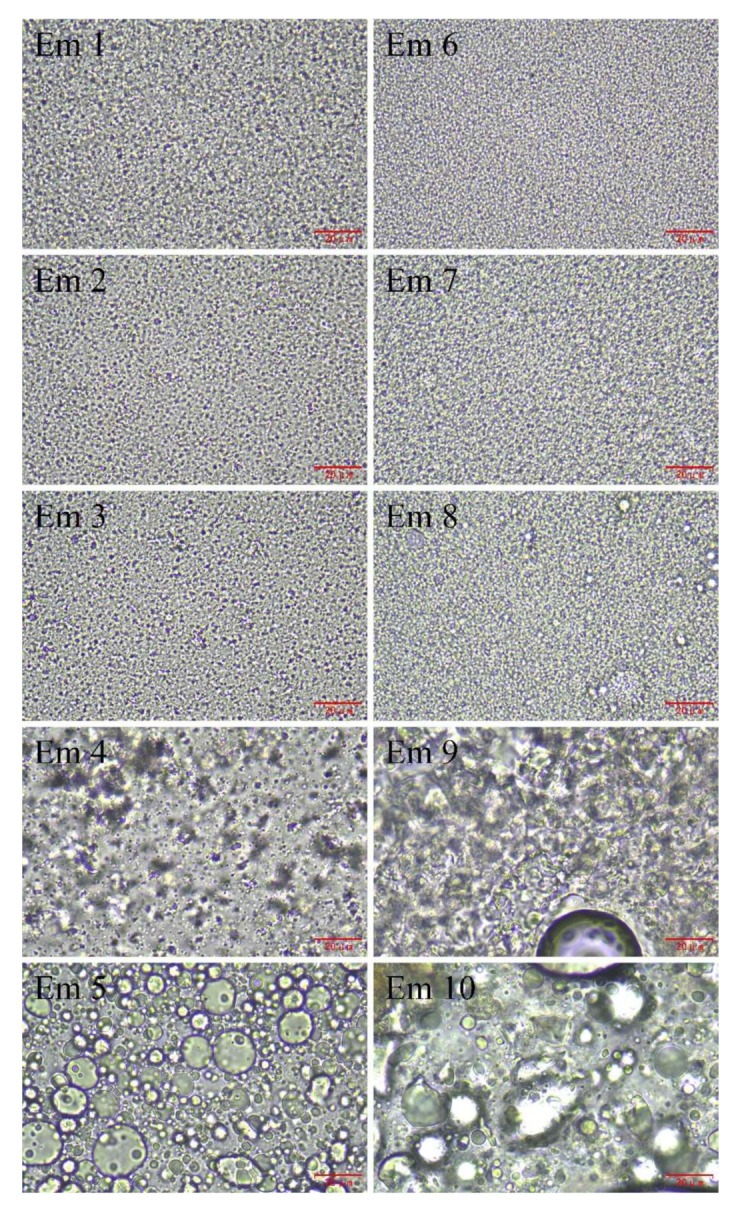
Optical microscopy images of W/O emulsions including Em 1, Em 2, Em 3, Em 4, Em 5, Em 6, Em 7, Em 8, Em 9, and Em 10. The scale bar is 20 μm in length.

Optical microscopy images of W/O emulsions are shown in Figure 4. Em 1, Em 2, Em 3, Em 6, Em 7, and Em 8 were composed of spherical droplets (Figure 4(Em 1–Em 3,Em 6–Em 8)). With the increase of lecithin in the proportion of the compound emulsifier to 1.5 w/% and 2.0 w/%, the stability of the emulsion decreased, and its microstructure also changed (Figure 4(Em 4,Em 5,Em 9,Em 10)). The gel-like structure was formed in the emulsion and gathered together, but the aggregation extent of olive oil-based emulsion was less than maize oil-based emulsion when the emulsifier ratio was 0.5 w/% PGPR and 1.5 w/% LE, as revealed with Em 4 and Em 9 (Figure 4(Em 4,Em 9)). This may be due to the difference in fatty acid composition between the two oils (Supplementary Table S1 in the Supplementary Materials), which leads to the difference in the formation of gel structure and aggregation extent. The research of Lindenstruth et al. indicated that the unsaturated fatty acids in the oil prevent the formation of a uniform film of lecithin at the oil-water interface [39]. There were non-spherical droplets in the emulsion, but there was a big difference between olive oil-based emulsion and maize oil-based emulsion when the emulsifier ratio was 0 w/% PGPR and 2.0 w/% LE, as revealed with Em 5 and Em 10 (Figure 4(Em 5,Em 10)). The droplets of the former are larger, but the gel structure was not observed, and the gel structure of the latter was still observed, and the system forms a bicontinuous phase due to the polymerization of droplets. The system showed high instability due to the flocculate and coalesce of the droplets when the proportion of lecithin in the emulsifier reached 1.5 w/% or more.

The above observation results are consistent with the observation results of Ushikubo et al., which indicated that lecithin with low-HLB alone cannot stabilize the W/O emulsion [2]. Previously, Knoth et al. observed a similar phenomenon when preparing W/O emulsions with lecithin and different oil phases (medium-chain triglyceride, sunflower oil, olive oil, butter oil, or MCT-oil/vegetable oil blends), and considered that the properties of lecithin-stabilized W/O emulsions were strongly dependent on the lipid type used as the continuous oil phase [4]. On the other hand, an emulsifier with suitable HLB is important to the morphology and stability of W/O emulsions. PGPR with low HLB and lecithin with high HLB can form emulsifiers with different HLB to adapt to the oil phase with different fatty acids composition to stabilize W/O emulsions.

### 3.6. Droplet Size Distribution Analysis of W/O Emulsions

**Figure 5:**
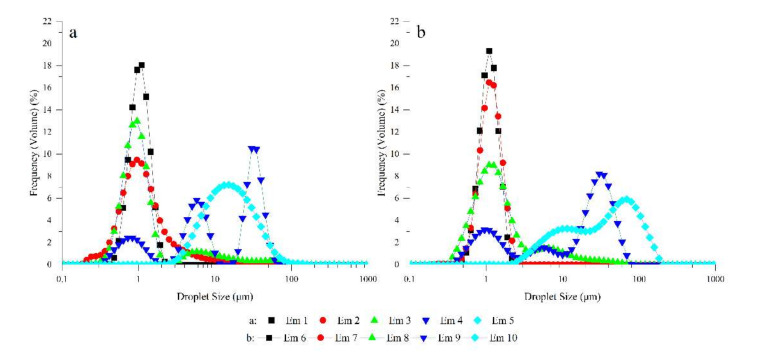
The droplet size distribution curve of W/O emulsions including Em 1 (■), Em 2 (●), Em 3 (▲), Em 4 (▼), and Em 5 (◆) in (**a**); Em 6 (■), Em 7 (●), Em 8 (▲), Em 9 (▼), and Em 10 (◆) in (**b**).

The initial droplet size distribution curves of all samples are shown in Figure 5. The uniformity of droplet size distribution and the Sauter mean diameter (d_3,2_) of samples are shown in Table 1.

The droplet size distribution curves of the two oil-based emulsions with high PGPR content and low LE content as an emulsifier showed a unimodal distribution, but the distribution characteristics were different. It is believed that emulsions with narrow symmetrical unimodal distribution and smaller Sauter mean diameter have better stability [40]. The droplet size distribution curve of Em 1 and Em 6, which contain 2.0 w/% PGPR and 0 w/% LE, exhibited a narrow unimodal distribution, and their Sauter mean diameter and symmetry were approximate. The droplet size distribution curve of Em 2 was wider than Em 7, while the Sauter mean diameter of Em 2 was smaller than Em 7. Although the droplet size distribution of Em 3 and Em 8 had a bimodal distribution, their Sauter mean diameters were also relatively small at 0.894 μm and 1.101 μm, respectively, since the principal part of the droplet size distribution was around 1.000 μm. The droplet size of the emulsion containing a higher content of PGPR is relatively small and uniform (Table 1), which is consistent with the results of optical microscopic observation (Figure 4(Em 1–Em 3,Em 6–Em 8)). The droplet size distribution curves of Em 4 and Em 9 reveal a trimodal distribution, but the former differs from the latter, which may be because there are fewer gel-like structures in olive oil-base emulsions than in corn oil-base emulsions. This is also the reason for the obvious increase in the Sauter mean diameter of the emulsion (Em 4 is 2.850 μm, Em 9 is 2.816 μm). This is consistent with the results of the optical microscopic observation (Figure 4(Em 5,Em 9)). Em 5 has a wide unimodal distribution because of droplet polymerization, which differs from the non-obvious bimodal distribution caused by gel structure aggregation of Em 10 and that the system exhibited instability.

### 3.7. Rheological Analysis of the W/O Emulsions

**Figure 6:**
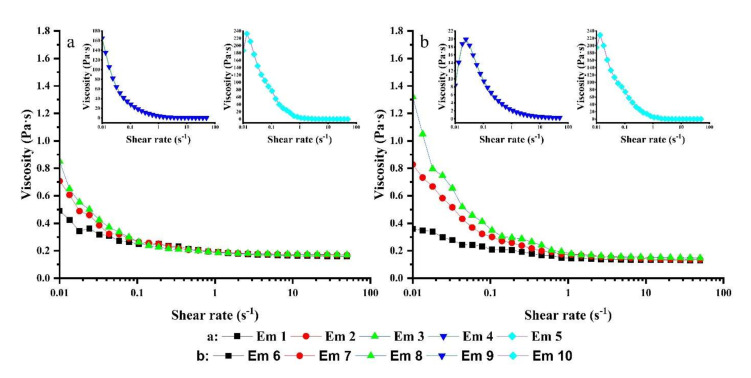
Viscosity (η) against shear rate (γ) curves of W/O emulsions including Em 1 (■), Em 2 (●), Em 3 (▲), Em 4 (▼), and Em 5 (◆) in (**a**); Em 6 (■), Em 7 (●), Em 8 (▲), Em 9 (▼), and Em 10 (◆) in (**b**).

The rheological properties of the W/O emulsions are revealed in Figure 6, and the static shear viscosity of W/O emulsions with different oil bases and proportions of compound emulsifiers are shown in Table 2.

Figure 6 indicated that all W/O emulsions have the characteristic of shear thinning, which is consistent with the phenomenon described by Hasan et al. [41]. It was previously reported that the rheological properties of the W/O emulsion system strongly depend on the rheological properties of the oil phase itself because the droplets formed by the water phase in the W/O emulsion only account for a small proportion in the system [42]. However, the differences between individual samples clarified that the influence of droplets on the rheological properties of the emulsion cannot be ignored. The appearance of shear thinning may also be attributed to the arrangement of droplets in the shear field, which provides a gradually reduced flow resistance [43]. With the increase in lecithin content, the trend of shear thinning became more obvious. The viscosity of W/O emulsions containing different oil bases with the same emulsifier composition were stable at a shear rate of about 1.00 s^−1^ and the trend of shear thinning was similar, except for Em 8 and Em 9. The curve of Em 8 displayed an upward trend, and the value of viscosity increased from 0.86 Pa·s to 0.92 Pa·s when the shear rate was from 0.04 s^−1^ to 0.08 s^−1^. The probable reason was that the droplets in the emulsion were polymerized or demulsified during shearing, resulting in a sudden change in viscosity. The stable point of Em 9 was about 15.00 s^−1^, which may be because the non-uniform droplet size of the sample is continuously rearranged under the action of shear force. With the increase in the proportion of lecithin in the emulsifier composition, the increase in the shear thinning trend of maize oil-based emulsions was significantly larger than olive oil-based emulsions.

Table 2 clarifies that the static shear viscosity of the emulsion increases significantly with the increase of lecithin ratio. On the one hand, the viscosity of the oil-water interface increases and the interaction between the droplets is enhanced, deriving from lecithin adsorbed on the oil-water interface, resulting in the increase of static shear viscosity. Part of the lecithin dissolves into the oil phase, resulting in an increase in the static shear viscosity of the oil phase. This could explain why the static shear viscosity increases with the increase of droplet size, which differs from the phenomenon that, with the decrease of droplet size, the increase of the specific surface area leads to the increase of friction and viscosity between droplets observed by Jinlong Li et al. [5]. In this experiment, Em 1 and Em 6 had smaller droplets (the Sauter mean diameter was 0.915 μm and 0.972 μm) and smaller static shear viscosity of 0.49 Pa·s and 0.82 Pa·s, respectively. Em 5 and Em 10 presented larger droplets (the Sauter mean diameter was 11.328 μm and the 15.968 μm) and larger static shear viscosity of 186.00 Pa·s and 556.80 Pa·s, respectively. Em 4, Em 5, Em 9, and Em 10 show greater static viscosity of 164.90 Pa·s, 186.60 Pa·s, 124.20 Pa·s, and 556.80 Pa·s, respectively, which may be caused by droplet polymerization and gel structure formation and aggregation in the emulsion.

### 3.8. Storage Stability Evaluation of the W/O Emulsions

**Figure 7:**
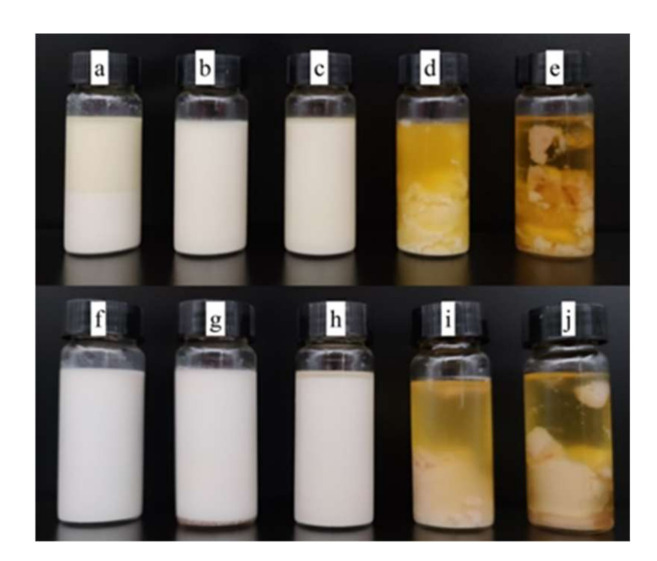
Digital photographs of W/O emulsions including Em 1 (**a**), Em 2 (**b**), Em 3 (**c**), Em 4 (**d**), Em 5 (**e**), Em 6 (**f**), Em 7 (**g**), Em 8 (**h**), Em 9 (**i**), and Em 10 (**j**), after 30 days of storage at room temperature.

**Figure 8:**
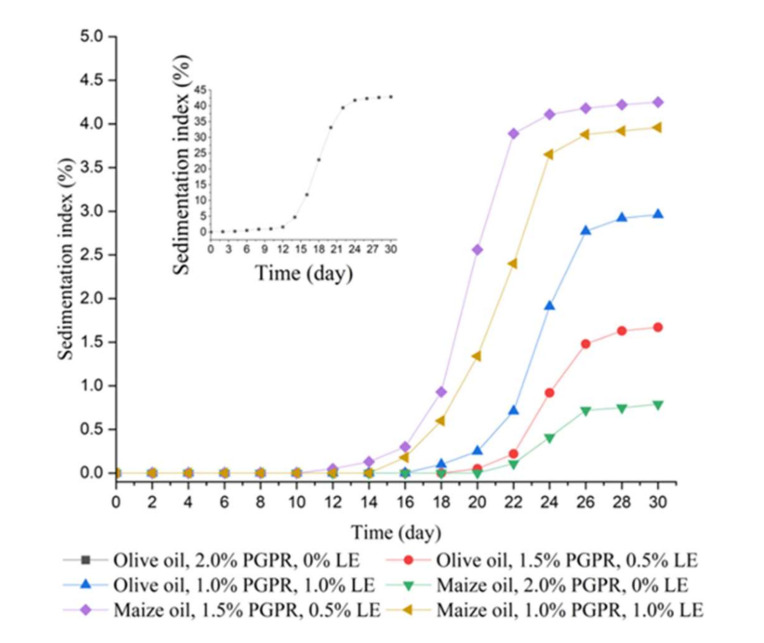
Variations in the sedimentation index of W/O emulsion including Em 1 (■), Em 2 (●), Em 3 (▲), Em 6 (▼), Em 7 (◆), and Em 8 (◀) during storage.

Figure 7 displays the apparent stability of W/O emulsions when stored at room temperature (25 °C) for 30 days, and the variation in sedimentation index (SI) of W/O emulsion with different oil bases and different emulsifier mass ratios during storage time is shown in Figure 8. It can be observed that Em 4, Em 5, Em 9, and Em 10 were unstable after preparation. Other emulsions remained stable since they presented no obvious phase separation within 10 days. Em 6 has the smallest SI of 0.79%, followed by Em 2, with a relatively small SI of 1.67%.

**Figure 9:**
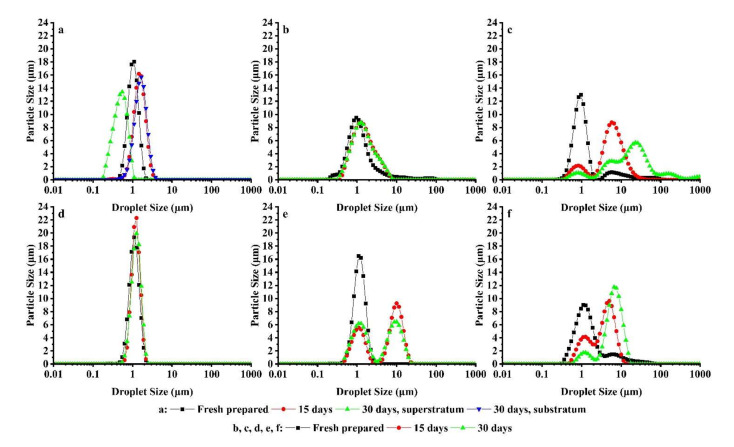
The droplet size distribution of W/O emulsions including Em 1 (**a**), Em 2 (**b**), Em 3 (**c**), Em 6 (**d**), Em 7 (**e**), and Em 8 (**f**), during storage. In addition, the test time includes fresh prepared (■), 15 days (●), 30 days superstratum (▲), 30 days substratum (▼) in a; fresh prepared (■), 15 days (●), 30 days (▲) in (**b**–**f**).

Droplet size distribution changes of the W/O emulsions during storage are revealed in Figure 9. Droplet size distribution curves shift in the direction of increasing droplet size with the increase of storage time and become nonuniformity, which is consistent with other literature reports [44,45,46]. The droplet size of the two-phase regions was measured, as revealed in Figure 9a, after the obvious phase separation of Em 1. The droplet size distribution of the upper phase region moved to the left side of the initial droplet size distribution, clarifying that the droplet size was smaller (*d*_3,2_ = 0.41 μm). The droplet size distribution in the lower phase region moves to the far right, showing that the droplet size in this part is larger (*d*_3,2_ = 1.367 μm). Em 6 has high storage stability and no significant deformation or deviation in the curve of droplet size distribution (Figure 9d). For Em 2, there was no obvious change in the droplet size distribution curve after 30 days of storage compared with that of 15 days of storage, clarifying that the emulsion system had good storage stability (Figure 9b). The droplet size distribution curve of Em 7 obviously changed from unimodal distribution to bimodal distribution and shifted to the right. In addition, it was noted that the height of the second peak of the droplet size distribution curve after 30 days of storage was lower than that of the droplet size distribution curve after 15 days of storage, and there was no significant difference between the first peak of the two curves. This may be due to the fact that the emulsion structure of some large droplets in the emulsion is destroyed and precipitated, resulting in a decrease in the number of droplets (Figure 9e). The droplet size distribution curves of Em 3 and Em 8 changed during storage, clarifying that the system was unstable.

The change in droplet size distribution can accurately reflect the microscopic changes and stability of the emulsion system during storage. The stability and practicability of the emulsion system can be comprehensively evaluated by the sedimentation index and the change of droplet size distribution. Em 2 and Em 6 have a smaller sedimentation index of 1.67% and 0.79%, respectively. There was only a slight change in the droplet size distribution curve during storage, and the Sauter mean diameter after 30 days of storage was 1.112 μm and 1.102 μm, respectively, which changed little compared with their corresponding initial values of 0.877 μm and 0.972 μm. To sum up, it is concluded that both have good stability and practical value. It is worth noting that the emulsifiers used in Em 2 and Em 6 are not the same. The emulsifier of Em 2 contains more lecithin than that of Em 6, so the HLB of the former is higher than that of the latter. We speculate that the emulsifier with low-HLB is suitable for stabilizing the W/O emulsion with olive oil, while the emulsifier with high-HLB is suitable for stabilizing the W/O emulsion with maize oil, because olive oil contains more MUFA and maize oil contains more PUFA. However, it has been reported that lecithin is difficult to stabilize W/O emulsion [4,47]. Our research indicates that lecithin has the potential to be used in combination with other low-HLB emulsifiers to stabilize the W/O emulsion.

## 4. Conclusions

In this study, water-in-oil (W/O) emulsions, which used olive oil and maize oil as the oil phase, loaded with sialic acid were established, and storage stability and stabilization mechanisms were clarified. The results displayed that sialic acid could combine with chitosan to form a stable gel network structure, which made the aqueous phase into a transparent colloidal state that prevented the occurrence of droplet polymerization and Ostwald ripening to enhance the stability of the emulsion. PGPR and LE were mixed in different proportions as co-emulsifiers to stabilize W/O emulsion and their increasing concentration presented different effects to the emulsion formation between the olive oil-base and the maize oil-base. The emulsifier with low-HLB is suitable for stabilizing the W/O emulsion with an oil phase containing more MUFA, while the emulsifier with high-HLB is suitable for stabilizing the W/O emulsion with an oil phase containing more PUFA. Finally, olive oil-based emulsions containing 1.5 w/% PGPR and 0.5 w/% LE and maize oil-based emulsions containing 2.0 w/% PGPR and 0 w/% LE showed the best performance. Initial droplet size distribution curves of stable emulsions displayed unimodal distribution, and the initial Sauter mean diameter is small (the former is 0.877 μm, the latter is 0.972 μm). The rheological curves displayed the characteristics of shear thinning and low static shear viscosity. In addition, storage stability was also examined, and an unsignificant change was found in droplet size distribution and Sauter mean diameter of the emulsions at room temperature (25 °C) for 30 days. These results may provide better knowledge of the utilization of sialic acid and would be interesting to expand its application in the food, cosmetic, and medical industries. On the other hand, it also provides a new reference for the construction of W/O emulsion with good stability and loading capacity based on different oil phases.

## Figures and Tables

**Table 1:** The Sauter mean diameter (d_3,2_) and uniformity of freshly prepared W/O emulsions with different oil base and emulsifier mass ratios.

PGPR (W/%)	LE (W/%)	Olive Oil		Maize Oil	
		D3,2 (Μm)	Uniformity	D3,2 (Μm)	Uniformity
2.0	0	0.915 ± 0.001	0.232 ± 0.001	0.972 ± 0.001	0.229 ± 0.002
1.5	0.5	0.877 ± 0.017	2.140 ± 0.145	0.998 ± 0.003	0.266 ± 0.003
1.0	1.0	0.894 ± 0.003	2.990 ± 0.266	1.101 ± 0.002	1.790 ± 0.026
0.5	1.5	2.850 ± 0.215	1.620 ± 0.047	2.816 ± 0.081	0.712 ± 0.017
0	2.0	11.328 ± 0.098	0.677 ± 0.015	15.968 ± 0.282	0.860 ± 0.019

**Table 2:** T Static shear viscosity of W/O emulsions with different oil bases and proportions of compound emulsifiers.

PGPR (W/%)	LE(W/%)	Olive Oil (Pa·S)	Maize Oil (Pa·S)
2.0	0	0.49	0.82
1.5	0.5	0.71	0.85
1.0	1.0	0.85	1.00
0.5	1.5	164.90	124.20
0	2.0	186.60	556.80

## Data Availability

The data that support the findings of this study are available upon request by contacting the corresponding author.

## Supplementary Materials

The following supporting information can be downloaded at: https://www.mdpi.com/article/10.3390/foods11060873/s1. Supplementary Table S1 Fatty acid composition of olive oil and maize oil.

## References

[1] Bande R.M., Prasad B., Mishra I.M., Wasewar K.L. (2008). Oil field effluent water treatment for safe disposal by electroflotation. Chem. Eng. J..

[2] Ushikubo F.Y., Cunha R.L. (2014). Stability mechanisms of liquid water-in-oil emulsions. Food Hydrocolloid..

[3] Chen B.C., Li H.J., Ding Y.P., Suo H.Y. (2012). Formation and microstructural characterization of whey protein isolate/beet pectin coacervations by laccase catalyzed cross-linking. Lwt.-Food Sci. Technol..

[4] Knoth A., Sdherze I., Muschiolik G. (2005). Effect of lipid type on water-in-oil-emulsions stabilized by phosphatidylcholine-depleted lecithin and polyglycerol polyricinoleate. Eur. J. Lipid. Sci. Tech..

[5] Li J.L., Qiao Z.H., Tatsumi E., Saito M., Cheng Y.Q., Yin L.J. (2013). A Novel Approach to Improving the Quality of Bittern-Solidified Tofu by W/O Controlled-Release Coagulant. 1: Preparation of W/O Bittern Coagulant and Its Controlled-Release Property. Food Bioprocess Tech..

[6] Wan J., Bick A., Sullivan M., Stone H.A. (2008). Controllable microfluidic production of microbubbles in water-in-oil emulsions and the formation of porous microparticles. Adv. Mater..

[7] Raviadaran R., Ng M.H., Manickam S., Chandran D. (2019). Ultrasound-assisted water-in-palm oil nano-emulsion: Influence of polyglycerol polyricinoleate and NaCl on its stability. Ultrason. Sonochem..

[8] Kralova I., Sjoblom J. (2009). Surfactants Used in Food Industry: A Review. J. Disper. Sci. Technol..

[9] Christiansen K. (2014). PGPR, Polyglycerolpolyricinoleate, E476.

[10] Atik D.S., Boluk E., Toker O.S., Palabiyik I., Konar N. (2020). Investigating the effects of lLecithin-PGPR mixture on physical properties of milk chocolate. Lwt.-Food Sci. Technol..

[11] Blix F.G., Gottschalk A., Klenk E. (1957). Proposed nomenclature in the field of neuraminic and sialic acids. Nature.

[12] Chen X., Varki A. (2010). Advances in the biology and chemistry of sialic acids. ACS Chem. Biol..

[13] Wright E.M., Loo D.D., Hirayama B.A., Turk E. (2004). Surprising versatility of Na+-glucose cotransporters: SLC5. Physiology.

[14] Zhang T., She Z., Huang Z., Li J., Luo X., Deng Y. (2014). Application of sialic acid/polysialic acid in the drug delivery systems. Sian. J. Pharm. Sci..

[15] Sato C., Kitajima K. (2013). Disialic, oligosialic and polysialic acids: Distribution, functions and related disease. J. Biochem..

[16] Tao F., Zhang Y., Ma C., Xu P. (2010). Biotechnological production and applications of N-acetyl-D-neuraminic acid: Current state and perspectives. Appl. Microbiol. Biotechnol..

[17] Traving C., Schauer R. (1998). Structure, function and metabolism of sialic acids. Cell Mol. Life Sci..

[18] Rohrig C.H., Choi S.S., Baldwin N. (2017). The nutritional role of free sialic acid, a human milk monosaccharide, and its application as a functional food ingredient. Crit. Rev. Food Sci. Nutr..

[19] Oetke C., Hinderlich S., Brossmer R., Reutter W., Pawlita M., Keppler O.T. (2001). Evidence for efficient uptake and incorporation of sialic acid by eukaryotic cells. Eur. J. Biochem..

[20] Rinaudo M. (2006). Chitin and chitosan: Properties and applications. Prog. Polym. Sci..

[21] Martinez A., Chornet E., Rodrigue D. (2004). Steady-shear rheology of concentrated chitosan solutions. J. Texture Stud..

[22] Kasaai M.R., Arul J., Charlet G. (2000). Intrinsic viscosity–molecular weight relationship for chitosan. J. Polym. Sci. Part B (Polym. Phys.).

[23] Mekhail M., Tabrizian M. (2014). Injectable Chitosan-Based Scaffolds in Regenerative Medicine and their Clinical Translatability. Adv. Healthc. Mater..

[24] Bhatia S., Bhatia S. (2016). Chitosan Based Nanomaterials and Its Applications. Systems for Drug Delivery: Safety, Animal, and Microbial Polysaccharides.

[25] Zhang K.M., Mao Z.J., Huang Y.C., Xu Y., Huang C.G., Guo Y., Ren X., Liu C.Y. (2020). Ultrasonic assisted water-in-oil emulsions encapsulating macro-molecular polysaccharide chitosan: Influence of molecular properties, emulsion viscosity and their stability. Ultrason. Sonochem..

[26] Rabelo C.A.S., Taarji N., Khalid N., Kobayashi I., Nakajima M., Neves M.A. (2018). Formulation and characterization of water-in-oil nanoemulsions loaded with acai berry anthocyanins: Insights of degradation kinetics and stability evaluation of anthocyanins and nanoemulsions. Food Res. Int..

[27] Luo S.Z., Hu X.F., Jia Y.J., Pan L.H., Zheng Z., Zhao Y.Y., Mu D.D., Zhong X.Y., Jiang S.T. (2019). Camellia oil-based oleogels structuring with tea polyphenol-palmitate particles and citrus pectin by emulsion-templated method: Preparation, characterization and potential application. Food Hydrocolloid..

[28] Amid B.T., Mirhosseini H. (2012). Emulsifying Activity, Particle Uniformity and Rheological Properties of a Natural Polysaccharide-Protein Biopolymer from Durian Seed. Food Biophys..

[29] Almeida M.L., Charin R.M., Nele M., Tavares F.W. (2017). Stability studies of high-stable water-in-oil model emulsions. J. Disper. Sci. Technol..

[30] Iqbal S., Xu Z.C., Huang H., Chen X.D. (2019). Structuring of water-in-oil emulsions using controlled aggregation of polysaccharide in aqueous phases. J. Food Eng..

[31] Sekeri S.H., Ibrahim M.N.M., Umar K., Yaqoob A.A., Azmi M.N., Hussin M.H., Othman M.B.H., Malik M.F.I.A. (2020). Preparation and characterization of nanosized lignin fromoil palm (Elaeis guineensis) biomass as a novel emulsifying agent. Int. J. Biol. Macromol..

[32] Ogutcu M., Arifoglu N., Yilmaz E. (2015). Preparation and Characterization of Virgin Olive Oil-Beeswax Oleogel Emulsion Products. J. Am. Oil. Chem. Soc..

[33] Kačuráková M., Wellner N., Ebringerová A., Hromádková Z., Belton P.S. (1999). Characterisation of xylan-type polysaccharides and associated cell wall components by FT-IR and FT-Raman spectroscopies. Food Hydrocolloid..

[34] Sorokin A., Lavlinskaya M. (2021). Synthesis of the superabsobents enriched in chitosan derivatives with excellent water absorption properties. Polym. Bull..

[35] Esfanjani A.F., Jafari S.M., Assadpoor E., Mohammadi A. (2015). Nano-encapsulation of saffron extract through double-layered multiple emulsions of pectin and whey protein concentrate. J. Food Eng..

[36] Yang J., Shen M.Y., Wu T., Luo Y., Li M.Y., Wen H.L., Xie J.H. (2020). Role of salt ions and molecular weights on the formation of Mesona chinensis polysaccharide-chitosan polyelectrolyte complex hydrogel. Food Chem..

[37] Hu X.Y., Wang Y.M., Zhang L.L., Xu M. (2019). Design of a novel polysaccharide-based cryogel using triallyl cyanurate as crosslinker for cell adhesion and proliferation. Int. J. Biol. Macromol..

[38] Hu X.Y., Wang Y.M., Zhang L.L., Xu M. (2020). Formation of self-assembled polyelectrolyte complex hydrogel derived from salecan and chitosan for sustained release of Vitamin C. Carbohyd. Polym..

[39] Lindenstruth K., Muller B.W. (2004). Parameters with influence on the droplet size of w/o emulsions. Pharmazie.

[40] Kowalska M. (2016). Physical Stability and the Droplet Distribution of Rice Oil-in-Water Emulsion. J. Disper. Sci. Technol..

[41] Hasan W., Khan M.N. (2020). Rheological characterization of vegetable oil blends: Effect of shear rate, temperature, and short-term heating. J. Food Process. Eng..

[42] Rietberg M.R., Rousseau D., Duizer L. (2012). Sensory Evaluation of Sodium Chloride-Containing Water-in-Oil Emulsions. J. Agric. Food Chem..

[43] Clements D. (2005). Food Emulsions: Principles, Practice and Techniques.

[44] Zafimahova-Ratisbonne A., Wardhono E.Y., Lanoiselle J.L., Saleh K., Clausse D. (2014). Stability of W/O Emulsions Encapsulating Polysaccharides. J. Disper. Sci. Technol..

[45] Esfanjani A.F., Jafari S.M., Assadpour E. (2017). Preparation of a multiple emulsion based on pectin-whey protein complex for encapsulation of saffron extract nanodroplets. Food Chem..

[46] Sommerling J.H., de Matos M.B.C., Hildebrandt E., Dessy A., Kok R.J., Nirschl H., Leneweit G. (2018). Instability Mechanisms of Water-in-Oil Nanoemulsions with Phospholipids: Temporal and Morphological Structures. Langmuir.

[47] Balcaen M., Steyls J., Schoeppe A., Nelis V., Van der Meeren P. (2021). Phosphatidylcholine-depleted lecithin: A clean-label low-HLB emulsifier to replace PGPR in w/o and w/o/w emulsions. J. Colloid. Interf. Sci..

