# Supplementary material for: Novel Water-in-Oil Emulsions for Co-Loading Sialic Acid and Chitosan: Formulation, Characterization, and Stability Evaluation
Source: Foods. 2022 Mar 18;11(6):873. doi: 10.3390/foods11060873 (PMC8951255; doi:10.3390/foods11060873)
Supplement: Supplementary file 1 [file foods-11-00873-s001.zip › foods-1626823-supplementary.pdf]

Supplementary Materials

Table S1. Fatty acids composition of olive oil and maize oil.

C chain	Fatty acid	Olive oil composition/%	Maize oil composition/
16:0	Palmitic acid	10.390	12.536
16:1	Palmitoleic acid	0.804	
18:0	Stearic acid	3.690	1.714
18:1	Oleic acid	73.323	30.356
18:2	Linoleic acid	10.030	53.244
18:3	Gamma linolenic acid		0.592
18:3	Alpha linolenic acid	0.720	0.686
20:0	Arachidic acid	0.419	0.404
20:1	Cis-11-Eicosenoic acid	0.262	0.335
22:0	behenic acid	0.164	
24:0	lignoceric acid		0.132
22:6	DHA	0.199	
	SFA	14.662	14.787
	UFA	85.338	85.213
	MUFA	74.389	30.691
	PUFA	10.948	54.522

Measurements:

The fatty acid composition of olive oil and maize oil was measured using a gas chromagraph (Agilent Technologies, 7890A GC system) equipped with a hydrogen flame detector.

50-55 mg of oil and 2 mL of 0.5 M sodium hydroxide methanol solution were added into a circular bottom centrifuge tube of 15mL, and reacted in boiling water until the grease disappears. Then, 2 mL of boron trifluoride methanol solution was added into the circular bottom centrifuge tube and continued to react in boiling water for 3 minutes. 2 mL of saturated NaCl solution and 2 mL of n-hexane would be poured into a circular bottom centrifuge tube after cooling at room temperature and mixed evenly by vortex oscillation. Absorb the Organic phase was filtered into the injection bottle with Organic filter membrane with pore size of 0.22 μm, and the samples were stored at -20 °C for gas chromatographic analysis.

Chromatographic column is DB-WAX capillary column (30 m × 0.25 mm × 0.25 μm). carrier gas is N_2 (purity ≥ 99.9%) and the flow rate was 0.8 mL/min. The temperature of injector and detector are 230 °C and 240 °C, respectively. The injection volume is 1 μL and split ratio is 50:1. The initial temperature of the oven is 60 °C and keep it for 2 minutes, then heated to 200 °C at 15 °C/min, and then heated to 230 °C at 3 °C/min and kept for 25 minutes.

The composition of different fatty acids in the product was calculated according to the Eq. (1).

$$\text{Fatty acid ratio (\%)} = \frac{\text{Peak area of fatty acid to be analyzed}}{\text{Peak area of all of fatty acid}} \times 100 \quad (1)$$
